# The Role of Risk Factor Modification in Atrial Fibrillation: Outcomes in Catheter Ablation

**DOI:** 10.3390/jcdd11040097

**Published:** 2024-03-25

**Authors:** Shahana Hussain, Neil Srinivasan, Syed Ahsan, Nikolaos Papageorgiou

**Affiliations:** 1 Electrophysiology Department, Barts Heart Centre, St Bartholomew’s Hospital, London EC1A 7BE, UK; shahana.hussain2@nhs.net (S.H.); syedyahsan@gmail.com (S.A.); 2 Department of Cardiac Electrophysiology, Essex Cardiothoracic Centre, Basildon SS16 5NL, UK; neil.srinivasan@nhs.net; 3 Circulatory Health Research Group, Medical Technology Research Centre, School of Medicine, Anglia Ruskin University, Chelmsford CM1 1SQ, UK; 4 Institute of Cardiovascular Science, University College London, London WC1E 6BT, UK

**Keywords:** atrial fibrillation, lifestyle modification, risk factors, catheter ablation, lifestyle, rhythm control, rate control

## Abstract

The management of atrial fibrillation has evolved significantly over the last ten years with advancements in medical and catheter ablation approaches, but these have limited success when used in isolation. Trends in the management of lifestyle modifications have surfaced, as it is now better understood that modifiable risk factors contribute significantly to the development and propagation of atrial fibrillation, as well as failure of treatment. International guidelines have integrated the role of lifestyle modification in the management of atrial fibrillation and specifically in the persistent form of atrial fibrillation; these guidelines must be addressed prior to considering catheter ablation. Effective risk factor modification is critical in increasing the likelihood of an arrhythmia-free survival following catheter ablation.

## 1. Introduction

Atrial fibrillation (AF) is the most encountered and sustained cardiac arrythmia, and its incidence is increasing on a global scale. AF is linked to an increase in all-cause mortality, stroke, heart failure, and recurrent hospitalisations [1]. Studies have shown a dramatic increase in the absolute number of incident AF by 72% over the last two decades due to a combination of increase in the number of older persons, comorbidity burdens such as obesity, sleep apnoea, hypertension, diabetes mellitus, etc., and a decline in the age of AF diagnosis, especially in males. It was also shown that in the most socioeconomically deprived individuals, the AF age at diagnosis was lower and there were a greater number of comorbidities. The increased disparity in socioeconomic gradients has a significant impact on the development and propagation of AF burden worldwide. It was also demonstrated that following AF diagnosis, cardiovascular mortality and hospitalisation declined [2,3].

These risk factors result in the structural and electrical remodelling of the left atrium (LA), leading to abnormalities in haemodynamics and electrophysiological properties of the atrial myocardium, resulting in an atrial cardiomyopathy. Once this has developed, it provides a substrate for the propagation and maintenance of AF. In time, paroxysmal AF will progress to the persistent form, and if risk factors are not managed and reversed, then ultimately progress to the permanent form of AF. Therefore, risk factor modification is key in the primary and secondary prevention of AF.

Evidence is gathered, showing that targeting these risk factors has an impact on the reduction in AF burden and the recurrence of atrial arrhythmias after ablation. Many international guidelines, including the European Society of Cardiology and the American Heart Association, strongly support risk factor modification as a critical component alongside anticoagulation, rate control, and rhythm control strategies in treating AF [4,5].

This review describes the current evidence into the association of different modifiable risk factors and their mechanisms on the progression of AF. It discusses the impact of the early management of these underlying conditions on AF outcomes, in combination with rhythm control strategies, to allow the implementation of a patient-tailored approach to AF management.

## 2. Early Detection and Treatment of Risk Factors

AF is thought to be a progressive disease with its standard trajectory developing from a paroxysmal self-terminating form with progression to persistent, and then long-standing persistent, and finally permanent AF [6,7]. Some patients already have persistent AF at the time of their first diagnosis. Data from registries show that patients with the persistent form of AF had a higher likelihood of being hospitalised due to cardiovascular causes and were noted to have a higher incidence of strokes, but they were also older and had a higher number of underlying co-morbidities, such as coronary artery disease, hypertension, and heart failure [7,8].

Many risk factors have been identified in the association of AF, such as hypertension, obesity, physical inactivity, excess alcohol consumption, and sleep apnoea [9,10,11,12,13,14]. There are also non-modifiable risk factors such as sex, ethnicity, genetic predisposition, and advancing age.

The underlying mechanisms for these risk factors driving AF have been explored in both animal and human studies. Ageing and hypertension were shown to result in remodelling of the LA, bi-atrial enlargement, increased atrial interstitial fibrosis, and a lower atrial effective refractory period (ERP) in mature, spontaneously hypertensive rats when compared to their normotensive counterparts [15]. Otsuka demonstrated that an increase in LA pressures, increased epicardial fat, and fatty infiltration were associated with an increased vulnerability to AF in the obese canine group, thus demonstrating the role of obesity in the pathogenesis of AF [16]. Studies have also described other mechanisms such as alterations in left atrial voltage, increased left ventricular hypertrophy, increased systemic vascular resistance, prothrombotic changes, and adverse changes in the renin angiotensin-aldosterone system (RAAS) as causative in AF [17,18,19,20,21].

Early detection of these underlying conditions and risk factor modification, ideally before AF clinically manifests, would reverse atrial re-modelling and prevent but also limit AF progression, as well as decrease the incidence of strokes and other cardiovascular adverse events [22,23]. Similarly, optimal control of these risk factors is associated with a reduced risk of AF recurrence post ablation.

## 3. Hypertension

The most prevalent modifiable risk factor for AF is hypertension, which accounts for 20% of cases [24]. It is linked with the dysregulation of the autonomic nervous system, altered haemodynamics, and activation of the RAAS system. Over time, uncontrolled hypertension results in inflammation and fibrosis, and ultimately the remodelling of the arteries, left ventricle, and the left atrium.

Chronic hypertension is strongly associated with the onset of AF, as well as its progression to persistent forms, and is an independent predictor of the recurrence of AF post ablation [7,25,26].

The Systolic Blood Pressure Intervention Trial (SPRINT) in 2014 was a randomised control trial of 9361 patients that compared two strategies for treating systolic BP. One strategy targeted the standard target of <140 mmHg and the other targeted <120 mmHg. The post hoc analysis of 8022 patients aged over 50 years without diabetes mellitus, heart failure, or previous stroke demonstrated that intensive BP control with a target of <120 mmHg was associated with a 26% lower risk of developing AF when compared to a target of <140 mmHg [27,28]; these findings were further validated by a meta-analysis [29].

Table 1 highlights relevant studies on the impact of hypertension and AF ablation outcomes. In a recent study across 55 centres in the German Ablation Registry consisting of 626 patients, the findings showed that the rate of AF recurrence, freedom of anti-arrhythmic drugs (AADs), and likelihood of repeat ablation were no different between patients who were diagnosed with hypertension versus those they were not diagnosed at the time of the ablation. However, they did note a higher incidence of angina and hospital admissions in those with hypertension [30]. Santoro further evaluated the impact of hypertension with AF ablation by recruiting 531 consecutive patients undergoing AF ablation. The study cohort included (1) patients with uncontrolled hypertension despite medical treatment *n* = 160), (2) patients with controlled hypertension (*n* = 192), and (3) patients without hypertension (*n* = 179). They concluded that hypertension itself was not correlated with an increased recurrence rate of AF following AF ablation, but patients in group 1—uncontrolled hypertension despite medical therapy pre-procedure—did lead to higher recurrence of AF (*p* = 0.045) [26].

The intensive management of isolated mild hypertension has not yielded a reduction in the recurrence of AF post ablation. The Substrate Modification with Aggressive Blood Pressure Control (SMAC-AF) trial was a randomised, open-label clinical trial which targeted blood pressures of <120 mmHg versus <140 mmHg in 184 patients undergoing AF ablation. They concluded there was no significant difference in AF recurrence or symptoms at 14 months’ median follow up [31].

The pathophysiological association between an increased sympathetic tone, AF, and hypertension has been seen in earlier studies. It had not been analysed in combination with pulmonary vein isolation (PVI) AF ablation. This led to the randomised study, ERADICATE-AF, which investigated the efficacy of renal denervation combined with PVI versus PVI alone in 302 paroxysmal AF patients with hypertension undergoing catheter ablation. They showed that freedom from AF, atrial flutter, or tachycardia at 12 months was significantly improved in the renal artery denervation group (72% versus 56%) without any increase in complications (*p* = 0.006). It is important to note that there was a lack of a formal sham-control renal denervation procedure in the control group [32].

It was established that renal denervation in addition to PVI reduced AF recurrence rates in hypertensive patients. In a recent study, Heradien et al. went on to explore whether renal denervation, without PVI, could prevent subclinical AF in patients with hypertensive heart disease. They undertook a randomised sham-controlled trial in 80 patients with uncontrolled hypertension taking 3 antihypertensive. The primary endpoint was the first subclinical AF episode, lasting >6 min and verified with an implantable cardiac monitor. They showed that the renal denervation group had a significantly lower incidence of subclinical AF compared to the control (19% versus 39.5%, *p* = 0.031). AF with vast ventricular response (>100 beats per min) also occurred significantly less frequently in the renal denervation group (2% versus 26%, *p* = 0.002) [33].

Recently, the Routine versus Aggressive risk factor driven upstream rhythm Control for prevention of Early atrial fibrillation in heart failure (RACE 3) study showed that targeted therapy of underlying conditions in patients with early persistent AF and mild to moderate heart failure (HF) was associated with a reduction in blood pressure, body mass index, lipid levels, and lowering of N-terminal pro-brain natriuretic peptide, indicating improvement of HF. On top of those beneficial effects on underlying conditions, this strategy improved the maintenance of sinus rhythm at the 1-year follow up [34].

## 4. Obesity

It has long been consistently established that an increased body mass index (BMI) is associated with an increased risk of AF in multiple cohort and case control studies as demonstrated in Table 2. The ARIC (Atherosclerosis Risk in Communities) study demonstrated that overweight and obesity (BMI > 25 kg/m^2^) accounted for 18% of incident AF, and obesity was the second strongest risk factor for the development of AF [35]. The Framingham Heart Study showed a 4% rise in AF risk for each unit increase in BMI [36].

Obesity is also an independent risk factor for recurrence in patients undergoing AF ablation, in particular at BMI > 30 kg/m^2^ where the risk of AF recurrence is at its highest [37], and therefore a focus should be on lifestyle modification to target obesity prior to undertaking an AF ablation.

In the landmark LEGACY (Long-Term Effect of Goal-Directed Weight Management in an Atrial Fibrillation Cohort: A Long-Term Follow-up Study) trial, Pathak established the significant impact of obesity and weight reduction and fluctuation on rhythm control. They recruited 1415 patients with persistent and paroxysmal forms of atrial fibrillation and a BMI of >27 kg/m^2^. The participants were offered a structured weight management programme including an exercise programme and regular meal plans. The final cohort consisted of 355 participants, and the weight loss was categorised into three groups: Group (1) ≥ 10% weight loss, Group (2) weight loss 3 to 9%, Group (3) < 3% weight loss. They demonstrated that AF burden was significantly reduced, and maintenance of sinus rhythm was higher in the groups with sustained weight loss (*p* < 0.001). This was particularly marked in the ≥10% weight loss group, where the participants had a six-fold higher likelihood of arrhythmia-free survival compared to the other two groups (*p* < 0.001) [38].

Data from a study of 701 patients with AF undergoing AF ablation with PVI showed that an increasing BMI had a positive correlation with obstructive sleep apnoea, hypertension, CHA_2_DS_2_-VASC score, and the occurrence of persistent AF (*p* ≤ 0.001). Radiofrequency ablation time also increased with BMI (*p* ≤ 0.001). Arrhythmia recurrence at 1 year was significantly higher with rising BMI categories, up to 57% in obese patients and 58% in morbidly obese patients (*p* = 0.007). Interestingly, the impact of an increasing BMI was not seen in the persistent AF population [39]. These results were further confirmed by a meta-analysis which found that every 5-unit increase in the BMI is associated with a 10% increase in post operative AF, but also 13% in AF recurrence post ablation [9]. The PREVEND study also echoed the finding that for every 5-unit increase in BMI, there was an increased rate of incident AF.

The SORT-AF (Supervised Obesity Reduction Trial) study was the first randomised multi-centre trial to evaluate the effect of a structured weight reduction programme on AF recurrence in obese patients undergoing AF ablation. They recruited patients with both paroxysmal and persistent AF, and all patients underwent a sleep apnoea screening and an implantable loop recorder insertion. Overall, the intervention cohort achieved a BMI decrease from a mean of 34.9 to 33.4 kg/m^2^ (average 4.6 kg weight loss), whereas the control group achieved a decrease in BMI from 34.8 to 34.5 kg/m^2^ (average 2 kg weight loss). Overall, AF burden decreased significantly (*p* = 0.001); however, there was no significant difference in AF recurrence reduction between the two groups. Further analyses did however show a significant association between BMI reduction and decreased AF recurrence for the persistent AF population [40].

These findings were not replicated in a further observational study of weight loss in 90 patients with long-standing persistent AF [41]. The lesion set involved pulmonary vein antral isolation, posterior wall isolation, and non-pulmonary vein trigger ablation. The weight loss programme included dietician-guided calorie restriction and a supported exercise programme. There was no improvement in AF recurrence or symptom severity at 12 months following a single AF ablation procedure. AF recurrence was monitored by Holter, an event recorder, and ECGs in both groups. There are multiple reasons why there may have been a failure of weight loss impact on AF burden in this population. Firstly, there is a reduced sensitivity of AF detection with intermittent cardiac monitoring, and an implantable loop recorder may have yielded a more accurate detection of AF burden. Secondly, this patient population has resistant AF due to the long-standing persistent nature with extra-pulmonary substrates requiring multiple ablation procedures.

The Swedish obese subject study was conducted in Sweden in patients with new-onset AF, and they concluded that bariatric surgery demonstrated a consistent and sustained weight loss (18% weight loss after 20 years follow up), and the risk of AF was reduced by 29% in the bariatric surgery group after 19 years follow up [42].

**Table 2:** Selected studies on impact of risk factor modification on weight loss and AF ablation outcomes.

Study	Year	Patient Number	Median Follow-Up (Months)	Study Design	Intervention	Results
ARREST-AF	2014	149	42	Prospective observational cohort study	Risk factor management was offered to patients with BMI > 27 kg/m^2^ and one other cardiac risk factor, after AF ablation. Target of weight loss > 10% or BMI < 27 kg/m^2^.	AF frequency, duration, symptoms, and symptom severity was significantly reduced in the risk factor management group (*p* < 0.001). Arrhythmia-free survival following a single procedure of AADs was also greater in the risk factor management group compared to the control group (*p* < 0.001) [43].
Glover et al.	2016	3333	12	Prospective observational cohort study	Evaluation of BMI impact on AF recurrence at 12 months following AF ablation	Patients with BMI ≥ 30 kg/m^2^ at baseline had a higher recurrence of AF following ablation [37].
Sivasambu et al.	2017	701	12	Prospective observational cohort study	Impact of obesity on patients undergoing PVI AF ablation.	Arrhythmia recurrence was significantly associated with increasing BMI with 39.9% in the control group with higher rates in all-high BMI groups (overweight, 51.3%; obese, 57%; morbidly obese, 58.1%; *p* = 0.007). Higher BMI was also associated with increased radiofrequency application time (*p* ≤ 0.001) and incidence of persistent AF (*p* ≤ 0.001) [39].
Mohanty et al.	2018	90	12	Prospective observational cohort study	Evaluation of weight loss interventions in patients with long-standing persistent AF versus control group undergoing AF ablation (PVI with posterior wall isolation and non-PV triggers).	Weight reduction improved quality of life but there was no improvement in symptom severity or long-term ablation outcomes. At 12 months follow up, 63.8% of patients in group 1 were arrhythmia free compared to 59.3% in group 2 off AADs (*p* = 0.68) [41].
SORT-AF	2021	133	12	Randomised control trial	Obese patients undergoing AF ablation were randomised to a structured weight loss programme or usual care to assess the impact of weight reduction on AF outcomes. All patients underwent OSA screening and ILR implantation.	The primary endpoint was AF burden between 3 and 12 months after ablation. Although AF burden decreased significantly after ablation, there was no significant difference between groups. Further subgroup analyses did show a significant correlation between BMI and AF recurrence for the persistent AF population (*p* = 0.032) [40]

Overall, this highlights the importance of targeted weight loss and lifestyle modification, especially in early onset AF, and this should be part of a comprehensive treatment approach to reduce AF burden but also to reduce the progression to persistent forms of AF.

## 5. Obstructive Sleep Apnoea and Ablation

Obstructive sleep apnoea (OSA) has become the focus in recent years as a novel risk factor for AF. The prevalence of OSA in patients with AF has been estimated to be as high as 50% [44]. Studies have shown patients with OSA are at a considerably higher risk of developing AF and it is an independent risk factor, particularly to those at the severe spectrum [45].

An observational study of 62 patients with polysomnography-confirmed OSA was undertaken to assess the impact of CPAP therapy on the recurrence rates of AF in those undergoing AF ablation with PVI. The study demonstrated that arrhythmia-free survival was higher in patients at 12 months treated with continuous positive airway pressure (CPAP) than patients that were not receiving CPAP (71.9% versus 36.7%, *p* = 0.01). These effects were sustained in arrhythmia-free survival off antiarrhythmic drugs (AADs) or in repeat ablation with a reduction in AF recurrence in patients receiving CPAP compared to the control (65.6% versus 33.3%, *p* = 0.02). The AF recurrence rate in CPAP-treated patients was similar to a group of patients without OSA [46]. This study once again demonstrates the importance of risk factor modification prior to undertaking catheter ablation.

Meta-analyses have predominantly utilised observational studies which have overall shown that CPAP treatment for AF patients with OSA might decrease the AF recurrence risks. A recent meta-analysis of 20 studies incorporating patient data from 54, 271 patients investigated the correlation of OSA and AF. They demonstrated that the incidence of AF was 88% higher in patients with OSA [47]. A subsequent meta-analysis of seven observational studies with a total of 4572 patients with AF after successful AF ablation were analysed. When compared to patients without OSA, the pooled odds ratio of recurrent AF in patients with OSA was 1.70 (95% CI, 1.40–2.06, I^2^ = 0). In patients with OSA, those who underwent successful catheter ablation, the use of CPAP showed a considerable reduction in risk of recurrence of AF with a pooled odds ratio of 0.28 (0.19–0.40, I^2^ = 0) [48].

Data from randomised controlled trials are limited, as shown in Table 3. A recent randomised control trial of 108 patients with paroxysmal AF and moderate to severe OSA was performed to assess the impact of CPAP on AF burden. Patients were randomised to receive 5 months of treatment with CPAP and with usual care versus usual care alone. AF burden was measured with an implantable loop recorder. Patients were followed up with for 5 months from treatment initiation. The study failed to show a protective effect for CPAP in the recurrence of AF [49]. This may be attributable to many limitations of the study; there was a short follow-up period of 5 months, a relatively small sample size, and a “lower than anticipated” percentage of time in AF, which reduced the power of the study.

A further randomised control trial (The SAVE study (Sleep Apnea Cardiovascular Endpoints)) did not show a reduction in incident AF with the use of CPAP in patients with moderate to severe OSA, with a background of coronary or cerebrovascular disease and with a primary composite cardiovascular endpoint (including AF). It is important to note that the study was underpowered, and the study design did not examine AF as a primary outcome [50]. A further randomised control trial assessing the impact of CPAP in patients with OSA and paroxysmal AF undergoing PVI ablation determined that, although PVI ablation did reduce the overall burden of AF in both cohorts, there was no statistical significance between groups [51]. This discrepancy in findings between observational studies and newer, randomised control trials may be due to observational studies employing older ablation technologies, which may impact outcomes.

It is key to recognise that OSA and AF share many characteristics—hypertension, obesity, age, diabetes—which are common to both conditions. Screening for OSA is vital when assessing patients with AF, as it should be a particular condition to other predisposing conditions such as obesity and diabetes. The ORBIT-AF study illustrated the beneficial use of CPAP in patients with AF and OSA, indicating reduced rates of progression to permanent AF [52].

**Table 3:** Selected studies on impact of risk factor modification on OSA therapy and AF ablation outcomes.

Study	Year	Patient Number	Median Follow Up (Months)	Study Design	Intervention	Results
Fein et al.	2013	62	12	Prospective observational cohort study	Evaluation of impact of CPAP therapy on AF recurrence in patients with polysomnography confirmed OSA undergoing AF ablation.	Patients receiving CPAP therapy had increased likelihood of freedom from AF/AT/AFL occurrence compared to patients that did not receive CPAP (71.9% vs. 36.7%, *p* = 0.01). The AF recurrence rate in the CPAP treated population was similar to patients without a diagnosis of OSA [46].
Congrete et al.	2018	4.572	12	Meta-analysis (7 observational studies)	Evaluation of AF recurrence in patients with OSA after AF ablation and the effect of CPAP on recurrence of AF.	AF recurrence was higher in patients with a diagnosis of OSA than without (pooled OR 1.70 (95% CI, 1.40–2.06)). The use of CPAP in patients with OSA was associated with a reduced risk of AF recurrence after catheter ablation (pooled OR of 0.28 (95% CI, 0.19–0.40)) [48].
Hunt et al.	2022	83	12	Randomised control trial	Impact of CPAP treatment on AF recurrence following PVI ablation in patients with PAF and OSA.	AF burden decreased in both cohorts but there was no significant different between groups (*p* = 0.69) [51].

## 6. Diabetes Mellitus and Ablation Outcomes

Several studies have demonstrated that diabetes mellitus and hyperglycaemia are significant risk factors for the development of AF [53,54]. Many studies have been shown to demonstrate the impact of both a diagnosis od diabetes mellitus and glycaemic control on AF ablation outcomes in Table 4. There have been many mechanisms proposed by which diabetes mellitus can promote AF; patients with AF have increased oxidative stress, glycaemic fluctuations, LV and wall thickness, pulse pressure, and stroke volume, which can lead to left atrial remodelling and atrial fibrosis, leading to increased arrhythmogenicity [54,55].

In a Danish nationwide cohort study, the risk for the development of AF was the highest in diabetic patients aged 18–39 years with an adjusted incidence rate ratio of 2.34 [56]. The ARIC study showed that diabetes and glycaemic control was an independent risk factor for incident AF [57]. At present, there are no guidelines supporting distinct HbA1c targets in patients with AF, more studies are needed to assess the benefit of strict glycaemic control and the recurrence of AF.

In patients undergoing AF ablation, raised hbA1c levels have been associated with an increased risk of AF recurrence [58]. Unsurprisingly, diabetes mellitus is also linked with an increased risk of AF recurrence post AF ablation in the persistent AF population [59]. In an observational study by Creta et al., which incorporated seven high-volume European centres with a total of 2504 patients undergoing AF ablation, the impact of type II diabetes mellitus was analysed. They noted that patients with diabetes mellitus tended to be older, had a higher BMI and had a higher prevalence of persistent AF. There was a higher recurrence of arrythmia 12 months post ablation in the diabetes mellitus cohort (32.0% versus 25.3%, *p* = 0.031). It is important to distinguish the subtype of AF, as persistent AF tends to be difficult to treat and often multiple AF ablations are required. Following the adjustment for the type of AF (paroxysmal versus persistent) over a median follow up of 17 ± 16 months, the results showed that atrial arrhythmia-free survival was significantly lower in the persistent AF population with diabetes (*p* = 0.003). These results were further validated in a propensity-matched analysis [60]. Diabetes mellitus was also an independent risk factor for AF recurrence on the multivariate analysis, which mirrors the results from previous studies.

It has been established that diabetes mellitus is an independent risk factor for the development of new-onset AF but also the recurrence of atrial arrhythmias following ablation. Studies have also looked into the effect of glycaemic control in diabetic patients undergoing AF ablation. Anselmino et al. performed a meta-analysis of fifteen studies inclusive of 1464 patients over a mean follow up of 27 months, assessing the efficacy of catheter ablation in patients with AF and diabetes mellitus and predictors of recurrence. Meta-regression analysis demonstrated that a higher body mass index (*p* < 0.001), higher basal glycated haemoglobin level (*p* < 0.001), and advanced age (*p* < 0.001) were associated with an increased incidence of arrhythmia recurrences [61].

A retrospective observational study of 298 patients evaluated the association between pre-AF ablation glycaemic control in diabetic patients over a mean follow-up period of 25.92 ± 20.26 months. They demonstrated that a higher glycated haemoglobin (HbA1c) positively correlated with higher recurrence rates of atrial arrythmias following ablation. Patients with a HbA1c value of >9% at the time of ablation were associated with a higher recurrence of AF (68.75%), as compared to patients with a HbA1c value of <7% who had a much lower risk of recurrence (32.4%, *p* = 0.0001). On multivariate analysis, the 12-month pre-ablation trend in HbA1c was significantly associated with AF recurrence with 10% improvement in HbA1c associated with only 2% recurrence. In patients with a worsening HbA1c trend 12 months prior to ablation, there was an associated increased risk of AF recurrence as high as 91% [62]. This demonstrates the importance of strict glycaemic control in those undergoing AF ablation.

**Table 4:** Selected studies on impact of risk factor modification in Diabetes Mellitus and AF ablation outcomes.

Study	Year	Patient Number	Median Follow-Up (Months)	Study Design	Intervention	Results
Anselmino et al.	2015	1464 patients	27 months	Meta analysis (15 studies)	Assess impact of diabetes mellitus on AF outcomes and predictors of AF recurrence	Higher basal glycated haemoglobin level was associated with a higher incidence of arrhythmia recurrences (*p* < 0.001) [61].
Donnellan et al.	2019	298	25.9	Prospective observational study	Impact of pre-ablation glycaemic control on AF recurrence rates	Arrhythmia recurrence was higher in patients with a higher HbA1c level, particularly those with values > 9 (*p* < 0.0001) [62].
Wang et al.	2020	351	29.5	Retrospective analysis	Comparison of AF outcomes in patients with and without DM	Arrhythmia recurrence was significantly higher in the group with DM (HR 2.24, 95% CI 1.42–3.55, *p* = 0.001) [59].
Creta et al.	2020	2504	17	Prospective observational study	Impact of type II diabetes mellitus on AF ablation outcomes and recurrence	Arrhythmia recurrence was higher 12 months post ablation in patients with DM than without (32.0% versus 25.3%, *p* = 0.031). Atrial arrhythmia survival was lower in patients with persistent AF and DM (*p* = 0.003) [60].

## 7. Alcohol Consumption

The impact of alcohol on the cardiovascular system has been well known and described in the literature, and there have been studies undertaken to evaluate the effect on clinical outcomes following AF ablation as demonstrated in Table 5. Heavy consumption over an extended period is associated with many harmful effects, including dilated cardiomyopathy. Ettinger first described the occurrence of cardiac arrhythmias in healthy people following acute ingestion of a large volume of alcohol known as ‘Holiday Heart syndrome’ in 1978, particularly AF. Since then, four decades have passed and research has shown the deleterious effects of excess alcohol on the cardiovascular system.

The Framingham Heart Study demonstrated that heavy alcohol consumption of >36 g/day was significantly associated with an increased risk of incident AF [63]. These results were further supported by outcomes from a prospective observation study of participants of the Swedish Inpatient Register, which concluded that alcohol consumption even at moderate amounts was a risk factor for the development of AF [64].

Effects of alcohol in patients undergoing AF ablation have shown that arrhythmia-free survival rates 12 months following AF ablation for paroxysmal AF are higher in patients who did not consume alcohol versus patients who did in a Japanese study (41.9% versus 34.1%; mean follow up, 44.4 ± 30.7 months; *p* = 0.003). Interestingly, there were no significant differences found in the LA size, duration of AF history, and incidence of non-pulmonary vein foci between the two groups (*p* = non-significant) [65].

Takahashi et al. studied the effects of alcohol abstinence on the reduction in AF burden following AF ablation in a multi-centre observational study of 1720 patients with paroxysmal (55.9%), persistent (31.6%), and long-standing persistent (12.5%) AF. Median baseline consumption was 140 g/week (interquartile range 70–280 g), which was decreased to a median of 70 g (interFquartile range 13–162 g) during the 1 year follow up. The study demonstrated that alcohol reduction of ≥1% from baseline to 1 year follow up was associated with a lower risk of atrial arrhythmia recurrence (HR 0.630, *p* < 0.001) [66]). The impact of alcohol abstinence on clinical outcomes following AF ablation was more pronounced in patients who consumed large quantities of alcohol before AF ablation.

Due to the limitation of studies regarding alcohol usage in comparison to other risk factors that can be objectively measured, the quantity of alcohol usage is self-reported by the individual and hence the reliance on self-reporting becomes a limiting factor. Barmano et al. assessed the impact of long-term alcohol consumption utilising ethyl glucuronide in hair (hEtG) as a biological marker in patients undergoing radiofrequency AF ablation. They examined potential associations with left atrial size and re-ablation within one year after the initial RFA. The study concluded that there was increased risk of AF recurrence in those who met or exceeded a 7 pg/mg cutoff, consistent with continuing alcohol consumption [67].

**Table 5:** Selected studies on impact of alcohol consumption and AF ablation outcomes.

Study	Year	Patient Number	Median Follow-Up (Months)	Study Design	Intervention	Results
Takigawa et al.	2016	1361	44	Prospective observational cohort study	Evaluation of impact of alcohol consumption on outcome of catheter ablation for paroxysmal AF.	The outcome between the two groups was similar (patients who consumed alcohol: 17.7% versus patients who did not consume alcohol 18.7%; *p* = 0.67). However, the frequency of alcohol consumption was significantly associated with AF recurrence after AF ablation (*p* = 0.04) [65].
Takahashi et al.	2021	1720	12	Prospective observational cohort study	Impact of alcohol abstinence on reduction in AF burden following AF ablation.	Alcohol reduction of ≥1% from baseline to 1-year follow up correlated with a lower risk of AF/AT recurrence (HR 0.630 [95% CI, 0.518–0.768], *p* < 0.001), as compared with alcohol reduction of <1% [66].
Barmano et al.	2019	192	12	Prospective observational study	Impact of alcohol intake using ethyl glucuronide in hair as a marker of long-term consumption, on AF ablation outcomes.	Male AF patients with hEtG ≥ 7 pg/mg, indicative of repeated alcohol consumption, experienced higher rates of re-ablation compared to patients with hEtG < 7 pg/mg (*p* = 0.017), although there was no significant different amongst females [67].

## 8. Implementation Strategies for Clinical Practice

The American College of Cardiology (ACC), American Heart Association (AHA), and Heart Rhythm Society (HRS) recently released the 2023 clinical practice guidelines for the management of AF, in which a new holistic approach to risk factor modification was introduced [68]. The authors recommended that patients are assessed in a structured “HEAD 2 TOES” approach. The HEAD 2 TOES risk factors are Heart failure, Exercise, Arterial hypertension, Diabetes type 2, Tobacco use, Obesity, Ethanol intake, and Sleep quality. Patients should be aided to adopt lifestyle changes to decrease the probability of developing AF, and in patients already diagnosed with AF, to reduce its burden and propagation into persistent and permanent forms of AF [69].

The “ABC pathway” is also an integrated management approach recommended by international guidelines that provides a clear strategy for clinicians; Avoid stroke, Better symptom management, and Cardiovascular and comorbidity risk reduction to deliver a holistic approach in the management of AF [70].

It is important to note that attention is now building on improving patient’s quality of life, not just AF burden; this recommendation focuses particularly on managing symptoms and allows the clinician to implement both rate and rhythm control strategies. Multiple studies have shown the implementation of the ABC pathway has been associated with an improved prognosis [69,70,71,72].

The emergence of digital health technologies allows clinicians and patients to improve overall AF management. Guo et al. demonstrated this by implementing the ABC approach using a mobile health technology-supported system, where cardiac rhythm monitoring was enabled through photoplethysmography (PPG)-smart devices, combined with patient’s self-reported symptoms and integrating information such as laboratory tests and co-morbidities to aide in the shared decision making. They demonstrated that this improved prognosis and reduced the rates of re-hospitalisation [73]. The findings were also substantiated by a further randomised control trial [74].

There are substantial trial data to support a structured and integrated approach to the management of AF where medical and procedural treatment options are combined with risk factor modification.

A comprehensive approach was shown to have the highest prospect of achieving an arrhythmia-free survival in patients post AF ablation, as shown in the ARREST-AF and LEGACY trials. The ARREST-AF study evaluated the effect of aggressive risk factor reduction in AF ablation outcomes. A total of 149 consecutive patients with a BMI ≥ 27 kg/m^2^ and ≥1 cardiac risk factor were recruited to the study. They were offered risk factor management (RFM) as per American Heart Association guidelines. Overall, 61 patients opted for RFM, and 88 patients declined, and they formed the control group. The patients were assessed 3–6 times monthly by clinical review and 7-day Holter monitoring. Patients in the RFM group underwent exercise stress testing, assessment of lipids, blood pressure, blood glucose levels, and a sleep apnoea assessment with overnight polysomnography, which were intensively managed. They also underwent lifestyle interventions such as dietary salt restriction, alcohol reduction, structured goal-directed weight reduction programme, and smoking cessation.

The study demonstrated a significant reduction In weight, blood pressure, improvements in glycaemic control and lipid profiles. At a mean follow up of 42 months, AF frequency, symptoms, duration, and symptom severity showed a significant reduction in the RFM group compared to the control (*p* = 0.001). There was a significant increased likelihood of drug-unassisted AF-free survival both after a single procedure and multiple procedures in the RFM group, with 16% requiring ongoing AADs, compared with 42% in the control. Arrhythmia-free survival in the RFM group overall was 87% compared to 17.8% in the control group [43].

Structured weight management programmes can be useful for certain individuals and, if necessary, bariatric surgery for morbidly obese patients, as data have shown results of a reduced AF burden and recurrence post ablation. A referral for overnight polysomnography should be considered if the patient is symptomatic but also if they are at risk with certain BMIs.

Alcohol and smoking reduction/cessation advice should also be given. Regarding physical activity initial targets of a 10% weight loss and a 2 MET increase in fitness can reduce the burden of AF. Over time, a target of BMI ≤ 25 kg/m^2^ can be aimed for along with 200 min per week of moderate-intensity activity. The use of smart watches, phone, and wearable devices has made these goals easier to objectively measure and achieve.

## 9. Risk Factor Modification Clinics

Multiple studies have consistently demonstrated the benefit of addressing the risk factors and therefore, the underlying substrate propagating the development and progression of AF as discussed above. Specialised AF clinics that focus on protocol-driven and guideline-directed treatment have proven to achieve a reduction in mortality and morbidity not only from AF, but overall cardiovascular disease [75]. Integrated multi-disciplinary clinics have also shown to increase patient compliance and improve clinical outcomes.

Vanharen et al. recently demonstrated the impact of nurse-led clinics, through a randomised control trial of 65 patients following ablation on AF recurrence, patient knowledge, lifestyle, and patient satisfaction. The patients were randomised to either standard care or standard care with additional nurse-led clinic intervention (an educational session, three consultations spread over 6 months, and telephone accessibility). The study demonstrated that at 6 months, AF recurrence was significantly lower in the intervention group (13.5 vs. 39.4%, *p* = 0.014). The intervention group also demonstrated increased patient satisfaction, physical activity, and a reduction in alcohol intake [76]. This demonstrates the importance of dedicated clinics in the diagnosis and management of modifiable risk factors to reduce AF burden, improve patient education, and reduce recurrence rates.

## 10. Discussion

Many modifiable risk factors significantly contribute to the development of AF both directly, and through the interlinking of metabolic conditions, such as diabetes and obesity. The importance of the recognition of these risk factors is critical in early lifestyle modification as a primary prevention of AF and the reduction in AF burden post ablation. The timely management of these conditions can significantly improve quality of life and AF burden.

An integrated, multi-disciplinary approach is needed to modify obesity, hypertension, OSA, alcohol use, diabetes mellitus, and tobacco use, this is also cost-effective long term.

The increasing global health burden of many of these conditions—obesity, diabetes etc—will result in an ongoing increase in AF burden worldwide, hence the urgent need for risk factor management clinics to manage the increasing burden of AF and reduce progression of AF to persistent and long-standing persistent subtypes of AF.

## Figures and Tables

**Table 1:** Selected studies on impact of risk factor modification on hypertension and AF ablation outcomes.

Study	Year	Patient Number	Median Follow Up (Months)	Study Design	Paroxysmal-AF Cases	Intervention	Results
German Ablation Registry	2020	626	42	Registry	64.5% in hypertensive group 72.5% in non-hypertensive group	Analysis of patients undergoing PVI AF ablation (RFA and cryoballoon was included) in patients with and without hypertension	Rates of AF recurrence, freedom from AADs and repeat ablation was not statistically significant amongst groups [30]
SMAC-AF	2017	184	14	Randomised open-label trial	57%	Participants assigned to aggressive BP target (<120/80) or standard BP (<140/90) prior to ablation. Primary outcome was >30 s of AF/AT/AFL occurrence	There was no significant difference between the groups (61.4% of aggressive BP control vs. 61.2% in standard treatment group) *p* = 0.763 [31]
Santoro et al.	2015	531	19	Prospective observational study	66%	Patients undergoing AF ablation in three groups: (1) Uncontrolled hypertension (2) Controlled hypertension (3) Without hypertension	Uncontrolled hypertension pre-procedure was associated with significant arrhythmia recurrence (*p* = 0.045), presence of non-PV triggers (58.8%, *p* < 0.001) as well as the presence of non-PAF (*p* = 0.002) [26]
ERADICATE-AF	2020	302	12	Randomised controlled trial	100%	Patients with hypertension and PAF were randomised to either PVI or PVI with renal denervation	Patients undergoing PVI with renal denervation had increased likelihood of freedom from AF/AT/AFL occurrence (72.1% vs. 56.5%, *p* = 0.006) [32]

## Data Availability

The data from this manuscript are derived from publicly available published clinical trial and study results.
